# Glances at the Hospitals

**Published:** 1900-09-15

**Authors:** 


### GLANCES AT THE HOSPITALS.

### THE ROCHDALE INFIRMARY AND DISPENSARY.

This institution presents its sixty-eighth annual report, from which we learn it contained 43 in-patients on January 1st, 1900. The admissions for the year numbered 374, of which 240 were cured, 62 were relieved, 9 left of their own accord, and 19 died. The daily number of beds occupied was 38, and the average duration of patients in the hospital was 34 days. The statistics of the out-patients' department show that 150 were under treatment at the beginning of the year, and that 2,521 new patients appeared. Of this total (2,671) 2,464 were discharged, 44 died, and 163 remained under treatment. The number of visits paid to patients at their homes was 2,919, the consultations were 12,165, and 48 patients were sent to the convalescent home. The total income was £2,944, and the expenditure was within a few pounds of this sum, but this satisfactory state of things was more apparent than real, because one legacy of £500 from Mr. Cheetham and another of £100 from Miss Barns had to be used as income instead of being invested, which, as the committee remark, is not altogether satisfactory, and points to the importance of increasing the annual subscriptions. These amounted to only £505, but the workmen's committee were able to hand over a sum of £610. A table showing the cause of death in every case is given, and another table shows the results obtained in the hospital, both in medical and surgical cases.

### THE SUSSEX COUNTY HOSPITAL AT BRIGHTON.

The seventy-second annual report shows that this hospital contains 190 beds, and that 1,677 in-patients were admitted during 1899. Of the total, 543 were discharged cured, 890 relieved, 99 for various other reasons, 115 died, and 157 remained under treatment on the date of the report. Of the deaths 28 took place within 24 hours of admission and 11 within 48 hours. Excluding these the death for the year was only 3·2 per cent. of the number treated. The out-patients reached the rather large total of 7,681, and we regret to notice that this was an increase of 695 on the number of the previous year; and that at the end of the year there were 2,102 out-patients on the books, showing an increase of 969. Judging from the abstract of the statutes incorporated with the statistics it does not appear that any wage limit is fixed by the governors above which gratuitous medical aid would be refused. If no such rule exists it would be most desirable to formulate one. Omitting legacies the ordinary receipts amounted to £9,602, being an increase of £1,490. The workmen's collections amounted to £540, but this was a decrease of nearly £20 on the collections for 1898. The ordinary expenditure was £12,681, showing an increase of £1,010. The cost of each in-patient averaged £6 7s. 3d., and each bed occupied £74 10s. 8d. Among the items of extraordinary expenditure were £3,300 transferred to the Special Improvement Fund, and £487 for new cooking apparatus. Mrs. Smith, of Arundel Terrace, Brighton, gave £1,000 to the Hospital, and Mr. Gassiot, of Upper Tooting, gave a like sum towards the expense of the new operating theatre. A convalescent home is attached to the infirmary, and 123 patients were sent there. There is also a nursing institute. Various medical and surgical tables are given, but they are not very well designed, and it is necessary to jump from one to another to obtain the mortality in any given operation. One table shows us that the operation for appendicitis was performed 17 times, and another shows that there were 2 deaths from this disease, and yet another shows that a total of 21 cases were under treatment; but whether the fatal cases were two of the 17 operated on, or two of the four not operated on, or one of each class, the tables are silent, at least so far as we understand them. Again we see that there were 10 cases of strangulated hernia, 8 operations and 2 deaths; but we are left in ignorance whether the latter are included in the 8 operations, or are the 2 not operated on. Altogether nearly 700 operations were performed during the year, and it seems a pity that such a rich field for teaching could not be utilised as a medical school. We hope that next year's report will show an improvement in the medical and surgical tables, and that a new one will be introduced showing the kind of anæsthetics used.

### THE GENERAL HOSPITAL AT TUNBRIDGE WELLS.

On January 1st, 1900, there were in residence 39 patients, admitted during the year 520, discharged recovered 372, relieved 99, unchanged 7, and died 35; remaining under treatment 46. The total number of out-patients was 4,469. Of these 2,379 recovered, 296 were relieved, 99 were given surgical appliances, 1,415 were treated for casualties, and 280 remained under treatment. The finances of the hospital were not so flourishing as might be desired at the end of 1898, for the accounts showed a deficit of £538, and it was deemed advisable to sell out railway stock to the extent of £715. Lately various legacies have been received, one from Mr. Debenham for £584, and another from Mrs. Jones for £45. A grant from the Marriott bequest for £3,000 was made by the Archbishop of Canterbury. During 1899 the ordinary receipts amounted to £3,353, and the expenditnre to £3,786, being nearly £300 lower than it was in the previous year. At the annual general meeting of the governors it was proposed by Dr. Ranking that a special committee, assisted by a hospital expert, be appointed to consider the question of the future administration of the hospital. This was carried unanimously. Presumably, therefore, changes in the administration are being contemplated. The house surgeon's report informs us that 203 medical cases were under treatment, and that 267 surgical operations were performed. We are left in ignorance as to the results of these operations, and the whole medical report is presented on one page. We trust that these statistics will be given more fully in subsequent reports.

### THE LIVERPOOL INFIRMARY FOR CHILDREN.

There were 1,005 in-patients and 13,969 out-patients treated during the year. An outbreak of diphtheria necessitated the closing of the infirmary for nearly three weeks, so that it might be thoroughly disinfected. Additional nurses have been engaged and a house obtained for them; but, of course, these alterations in the staff will entail considerable extra expenditure, and the committee's appeal for more funds is not unreasonable. It seems also that both city and poor rates are demanded. The committee very truly observe that this charge appears most unjust, and so it is, considering the amount which the hospital saves the rates. All this comes at an unfortunate time, as the annual subscriptions show a tendency to fall off. Of the 1,005 in-patients, 595 were discharged cured, 220 relieved, 20 not relieved, 61 for other reasons, and 107 died. The medical tables are very carefully compiled, and can be followed without difficulty. There were 409 operations performed on the in-patients. That for the radical cure of hernia, four times, all successful; for strangulated hernia, five times, with one death. Taking the total of the 409 operations there were 23 deaths, 328 recoveries, 56 relieved, and 2 not relieved. Among the out-patients 1,119 operations were performed, and only three deaths resulted. We should like to suggest that a table be inserted next year showing the anæsthetics used.

